# Tau pathology as determinant of changes in atrophy and cerebral blood flow: a multi-modal longitudinal imaging study

**DOI:** 10.1007/s00259-023-06196-2

**Published:** 2023-03-28

**Authors:** Denise Visser, Sander C. J. Verfaillie, Iris Bosch, Iman Brouwer, Hayel Tuncel, Emma M. Coomans, Roos M. Rikken, Sophie E. Mastenbroek, Sandeep S. V. Golla, Frederik Barkhof, Elsmarieke van de Giessen, Bart N. M. van Berckel, Wiesje M. van der Flier, Rik Ossenkoppele

**Affiliations:** 1 Department of Radiology & Nuclear Medicine, Amsterdam Neuroscience, Vrije Universiteit Amsterdam, Amsterdam UMC, P.O. Box 7057, 1007 MB Amsterdam, The Netherlands; 2 Amsterdam Neuroscience, Neurodegeneration, Amsterdam, The Netherlands; 3 Amsterdam Neuroscience, Brain Imaging, Amsterdam, The Netherlands; 4 Medical Psychology, Amsterdam UMC Location University of Amsterdam, Meibergdreef 9, Amsterdam, the Netherlands; 5 Department of Psychiatry and Neurochemistry, Institute of Neuroscience and Physiology, The Sahlgrenska Academy, University of Gothenburg, Gothenburg, Sweden; 6 Wallenberg Centre for Molecular and Translational Medicine, University of Gothenburg, Gothenburg, Sweden; 7 Clinical Memory Research Unit, Lund University, Lund, Sweden; 8 Institutes of Neurology and Healthcare Engineering, University College London, London, UK; 9 Alzheimer Center Amsterdam, Department of Neurology, Amsterdam Neuroscience, Vrije Universiteit Amsterdam, Amsterdam UMC location VUmc, Amsterdam, The Netherlands; 10 Department of Epidemiology and Data Science, Vrije Universiteit Amsterdam, Amsterdam UMC, Amsterdam, The Netherlands

**Keywords:** Tau PET, Cerebral blood flow, Atrophy, Alzheimer’s disease, Longitudinal

## Abstract

**Purpose:**

Tau pathology is associated with concurrent atrophy and decreased cerebral blood flow (CBF) in Alzheimer’s disease (AD), but less is known about their temporal relationships. Our aim was therefore to investigate the association of concurrent and longitudinal tau PET with longitudinal changes in atrophy and relative CBF.

**Methods:**

We included 61 individuals from the Amsterdam Dementia Cohort (mean age 65.1 ± 7.5 years, 44% female, 57% amyloid-β positive [Aβ +], 26 cognitively impaired [CI]) who underwent dynamic [^18^F]flortaucipir PET and structural MRI at baseline and 25 ± 5 months follow-up. In addition, we included 86 individuals (68 CI) who only underwent baseline dynamic [^18^F]flortaucipir PET and MRI scans to increase power in our statistical models. We obtained [^18^F]flortaucipir PET binding potential (BP_ND_) and *R*_1_ values reflecting tau load and relative CBF, respectively, and computed cortical thickness from the structural MRI scans using FreeSurfer. We assessed the regional associations between i) baseline and ii) annual change in tau PET BP_ND_ in Braak I, III/IV, and V/VI regions and cortical thickness or *R*_1_ in cortical gray matter regions (spanning the whole brain) over time using linear mixed models with random intercepts adjusted for age, sex, time between baseline and follow-up assessments, and baseline BP_ND_ in case of analyses with annual change as determinant. All analyses were performed in Aβ−  cognitively normal (CN) individuals and Aβ+  (CN and CI) individuals separately.

**Results:**

In Aβ+ individuals, greater baseline Braak III/IV and V/VI tau PET binding was associated with faster cortical thinning in primarily frontotemporal regions. Annual changes in tau PET were not associated with cortical thinning over time in either Aβ+ or Aβ−  individuals. Baseline tau PET was not associated with longitudinal changes in relative CBF, but increases in Braak III/IV tau PET over time were associated with increases in parietal relative CBF over time in Aβ + individuals.

**Conclusion:**

We showed that higher tau load was related to accelerated cortical thinning, but not to decreases in relative CBF. Moreover, tau PET load at baseline was a stronger predictor of cortical thinning than change of tau PET signal.

**Supplementary Information:**

The online version contains supplementary material available at 10.1007/s00259-023-06196-2.

## Introduction

Histopathological and in vitro studies have shown that tau pathology is closely associated with neuronal injury (synaptic alterations and neuronal loss) in Alzheimer’s disease (AD) [1–6]. The positron emission tomography (PET) tracer [^18^F]flortaucipir binds to paired helical filaments of tau and has enabled examination of the relationship between tau pathology and neuronal injury or neurodegeneration in vivo in AD [7–10]. In line with histopathological and in vitro studies, neuroimaging studies have demonstrated strong correlations between baseline tau PET with cross-sectional atrophy in AD patients ([1, 10, 11]). In addition, longitudinal studies with relatively short follow-up time (i.e., 12–15 months) have shown that tau load also predicts future atrophy rates [2, 5, 11–13]. To better understand how tau PET and neurodegeneration are related, it is important to study their dynamic associations over time and investigate how baseline tau load and change in tau load associate with longitudinal cortical thinning.

Another aspect of neuronal injury in AD is the progressive reduction of cerebral blood flow (CBF). Dynamic scanning protocols can be utilized to obtain a measure of *R*_1_ [14, 15]. R_1_ is a proxy for relative cerebral blood flow (rCBF) and is closely associated with metabolic activity ([^18^F]FDG PET) and ^15^O-H_2_O PET (i.e., the “gold standard” for measuring flow) [16–18]. Previous studies using other imaging techniques such as arterial spin labeling (ASL) to measure CBF have demonstrated decreased CBF (or cerebral perfusion) in (probable) AD patients [19, 20]. When it comes to the relationship between tau pathology and CBF (cross-sectional), it has been shown that higher levels of tau pathology are associated with locally decreased rCBF in AD [14, 21]. Taken together, these studies indicate that baseline tau pathology is related to neuronal injury as reflected by decreased CBF as well as atrophy in AD. However, it is less well established whether (rate of) change in tau pathology also relates to (rate of) change in CBF.

With respect to the order of pathophysiological processes in AD, general consensus has been reached for accumulation of tau pathology (relatively early event) and atrophy (relatively late event) [4, 22]. There is less agreement regarding rCBF changes, as some studies suggest CBF to be an early biomarker of disease [19], while others suggest changes in CBF to potentially be both cause and consequence of protein accumulation [23]. Many studies suggested that neuronal injury imaging markers change relatively late in the disease process, after the first observation of protein accumulation (such as amyloid or tau pathology) on PET or in CSF [4, 21, 22, 24]. The aim of this longitudinal study was to investigate the associations between changes in tau PET with imaging biomarkers of neuronal injury (i.e., atrophy and CBF) in a cohort comprising i) amyloid negative (Aβ−) cognitively normal (CN) individuals, and ii) amyloid positive (Aβ+) CN and cognitively impaired (CI) (AD-phenotype) individuals. First, we assessed whether tau PET, atrophy, and rCBF showed significant changes over time. Second, we assessed the association between (i) baseline and (ii) annual change in tau PET (binding potential [BP_ND_]) and longitudinal changes in both cortical thickness and rCBF (*R*_1_). Assuming that CBF alterations occur in between tau accumulation and atrophy in the pathophysiological development of AD, we hypothesized that higher tau load at baseline would be strongly associated with a steeper decline in cortical thickness and rCBF in Aβ+ individuals. Furthermore, we hypothesized that larger increases in tau PET over time would be associated with larger decreases in cortical thickness and rCBF in Aβ+ individuals.

## Methods

### Participants

We included 61 individuals from the Amsterdam Dementia Cohort (ADC) of the Alzheimer Center Amsterdam [25] of whom 26 were CN Aβ− with subjective cognitive decline (SCD) [26] and 35 Aβ+ with SCD (CN, *n* = 9), or cognitively impaired AD (CI, *n* = 26) [27, 28]. All 61 participants underwent baseline and 2-year follow-up dynamic [^18^F]flortaucipir PET and MRI scans, and are referred to as the follow-up sample. In addition, to increase power in our statistical models, we included 86 individuals from the ADC (18 CN and 68 CI) who only underwent baseline dynamic [^18^F]flortaucipir PET and MRI scans, referred to as the baseline-only sample. Individuals with SCD (n_follow-up + baseline-only_ = 55) were recruited from the SCIENCe cohort [29], which is part of the ADC [25]. All individuals underwent a standardized diagnostic workup, including medical and neurological examination, assessment of vital functions, informant-based history, neuropsychological evaluation, magnetic resonance imaging (MRI), and standard labs. Subsequently, diagnoses were determined by consensus in a multidisciplinary meeting. AD biomarkers in cerebrospinal fluid (CSF) and/or Aβ PET were available for all individuals. Individuals were classified as Aβ+ based on abnormal AD biomarkers (CSF Aβ42 <813 pg/mL [30] and/or abnormal Aβ PET (on visual read)). When both CSF Aβ42 and Aβ PET were available, Aβ PET was used for the determination of Aβ status. All AD patients had abnormal AD biomarkers and are therefore considered in the AD pathophysiological continuum, according to the NIA-AA Research Framework, classified as Aβ+ CI individuals [22]. Individuals with SCD with evidence of Aβ pathology were classified as Aβ+ CN individuals. Amyloid-β status was only determined at baseline. Exclusion criteria included severe traumatic brain injury, abnormalities on MRI likely to interfere with segmentation of tau PET and participation in a drug trial with tau or Aβ-targeting agents. Individuals were grouped based on amyloid status (negative and positive) for analyses. The study is in accordance with the ethical standards of the Medical Ethics Review Committee of the Amsterdam UMC VU Medical Center and with the 1964 Helsinki Declaration and its later amendments. Written informed consent was obtained from all participants prior to participation.

### Image acquisition

All 61 individuals from the follow-up sample underwent dynamic [^18^F]flortaucipir PET and MRI scans at baseline and 2-year follow-up. Dynamic [^18^F]flortaucipir PET scans were acquired on a PET-CT scanner (Philips Medical Systems, Best, The Netherlands) at the Amsterdam UMC VU Medical Center. Individual doses of [^18^F]flortaucipir were synthesized on site, using a previously described protocol [31]. All individuals at baseline participated in an initial scanning protocol of 130 min, consisting of a 60-min dynamic emission scan, a 20-min break, and another dynamic emission scan between 80 and 130 min post-injection [31]. In order to lower the burden associated with a long scanning protocol, especially for AD patients, our research group recently validated a quantitatively accurate shortened scanning protocol of 100 min [32]. Subsequently, all CI AD patients participating in follow-up PET scans for our ongoing longitudinal cohort study participated in the recently validated scanning protocol of 100 min, consisting of a 30-min dynamic emission scan, a 50-min break, and a second dynamic emission scan between 80 and 100 min post-injection. In order to correct for this adjusted scanning protocol at follow-up, the 130-min baseline scans of these individuals were analyzed as biphasic 100-min scans (*n* = 26). Scanning protocols were initiated with a low-dose CT for attenuation correction, followed by simultaneously injecting  ~240 ± 10 MBq [^18^F]flortaucipir (bolus) and starting the first dynamic emission scan. After a break and a second low-dose CT for attenuation correction, another dynamic emission scan was performed. During scan procedures, head movements were restricted by a headband and head positioning was regularly checked using laser beams. PET scans were reconstructed with a matrix size of 128 × 128 × 90 and a voxel size of 2 × 2 × 2 mm^3^, including standard corrections for attenuation, dead time, randoms, decay, and scatter. For each scan protocol, the later dynamic PET scan was coregistered to the first dynamic PET scan into a single dataset. Furthermore, all individuals underwent a structural whole-brain MRI scan on a 3.0 Tesla (3 T) MRI scanner at baseline and follow-up (Ingenuity Time-of-Flight (Phillips medical systems, Best, The Netherlands)). The scanning protocol for the Ingenuity Time-of-Flight scanner included an isotropic structural 3D T1-weighted image using a sagittal turbo gradient-echo sequence (1.00 m^3^ isotropic voxels, repetition time = 7.9 ms, echo times = 4.5 ms and flip angle = 8°) and a 3D fluid-attenuated inversion recovery (FLAIR) image (1.04 × 1.04 × 1.12 mm voxels, repetition time = 4800 ms, echo time = 278.8 ms, flip angle 90°). The 86 individuals from the baseline-only sample only underwent the above-described dynamic [^18^F]flortaucipir PET and MRI scans at baseline.

### PET image analysis

PET image analysis has been described previously [14, 31, 32]. Briefly, individual T1-weighted MRI scans were co-registered to native PET space, using Vinci software (Max Plank Institute, Cologne, Germany). The Hammers and Svarer templates incorporated in PVElab software were used to define cortical gray matter regions of interest (ROIs) on the co-registered MRI scans [33, 34]. Receptor parametric mapping (RPM) was applied to the PET data to obtain parametric images of BP_ND_ and R_1_, with the cerebellum gray matter as a reference region [35]. Previous research from our group has demonstrated that RPM is the best parametric method for [^18^F]flortaucipir [36], and has an excellent test-retest repeatability [9]. Tau PET data were additionally partial volume corrected using Van Cittert iterative deconvolution methods (IDM), combined with highly constrained back-projections (HYPR) as described previously [14]. Since results remained essentially unchanged when using partial-volume corrected (PVC) data, only non-partial volume corrected data are presented throughout the manuscript. We obtained BP_ND_ values (bilateral volume-weighted average) in three a priori defined regions corresponding to Braak staging regions of tau pathology (Braak I, Braak III/IV, and Braak V/VI), as described previously [10]. For *R*_1_ we used all cortical gray matter ROIs as available in the Hammers template, with addition of the entorhinal ROI from the Svarer template.

### MR image analysis

Cortical thickness (in mm) was obtained using FreeSurfer version 6.0.1 (https://surfer.nmr.mgh.harvard.edu/). MR images from individuals with longitudinal data were processed through the recon-all longitudinal processing stream, including motion correction, skull-stripping, registration, segmentation, smoothing, and parcellation mapping [37]. Images from individuals who only underwent baseline scans were processed through the recon-all processing stream for single timepoints. For both processing streams, cortical parcellation was performed based on the Desikan-Killiany Atlas (DKT), which contains 34 regions per hemisphere [38]. In order to improve cortical segmentation, a combination of T1- weighted+FLAIR images was used as input [39]. For individuals with missing FLAIR data at baseline and/or follow-up, only T1-weighted images were used for cortical segmentation at all available time points (*n*_Aβ−_  = 1, *n*_Aβ+_  = 3 out of follow-up sample (n_total_ = 61); *n*_Aβ+_  = 5 out of baseline-only sample (*n*_total_ = 86)). Following all processing streams, segmentation, and parcellation qualities were manually inspected for gross abnormalities. For cortical thickness, we used all cortical gray matter ROIs as available in the DKT atlas.

### Statistical analysis

All analyses were performed for Aβ+ and Aβ− individuals separately. To establish whether imaging markers changed over time, we performed linear mixed effects models (LMMs) with global BP_ND_, *R*_1_, or cortical thickness as dependent variable and time as determinant, adjusted for age-at-PET and sex (using both the follow-up and baseline-only sample). To assess differences in demographic variables and BP_ND_, R_1_, and cortical thickness, between Aβ− and Aβ+ individuals, two-sample *t*-tests were used. Associations between baseline BP_ND_ in our three regions of interest (Braak I, Braak III/IV, and Braak V/VI) and cortical thickness or R_1_ over time (in all cortical regions available in the brain templates) were assessed using LMMs with random intercepts and fixed slopes, adjusted for age, sex and time between baseline and follow-up assessments. For these analyses individuals from both the follow-up and baseline-only samples were used (*n*_total_ = 147). Next, the associations between annual change in BP_ND_ and longitudinal cortical thickness or R_1_ were assessed using the same LMMs, now with annual change in BP_ND_ as predictor and additionally adjusted for baseline BP_ND_. Therefore, annual change for BP_ND_ was calculated for all three regions of interest (Braak I, III/IV, and V/VI) by subtracting the baseline value from the follow-up value and dividing by the time between measurements in years. For these analyses, only individuals from the follow-up sample were used (*n* = 61). Results are reported both with and without the Benjamini-Hochberg False Discovery Rate (FDR) correction with a *Q* value of 5%. All statistical analyses were performed in Rstudio v4.0.3 and results are visualized using forest plots of the effect sizes and their respective confidence intervals. The regional associations are displayed using the ggseg R package for FDR-surviving cortical thickness ROIs only (since ggseg does not yet support the atlases used for *R*_1_).

## Results

### Participants

**Fig. 1:**
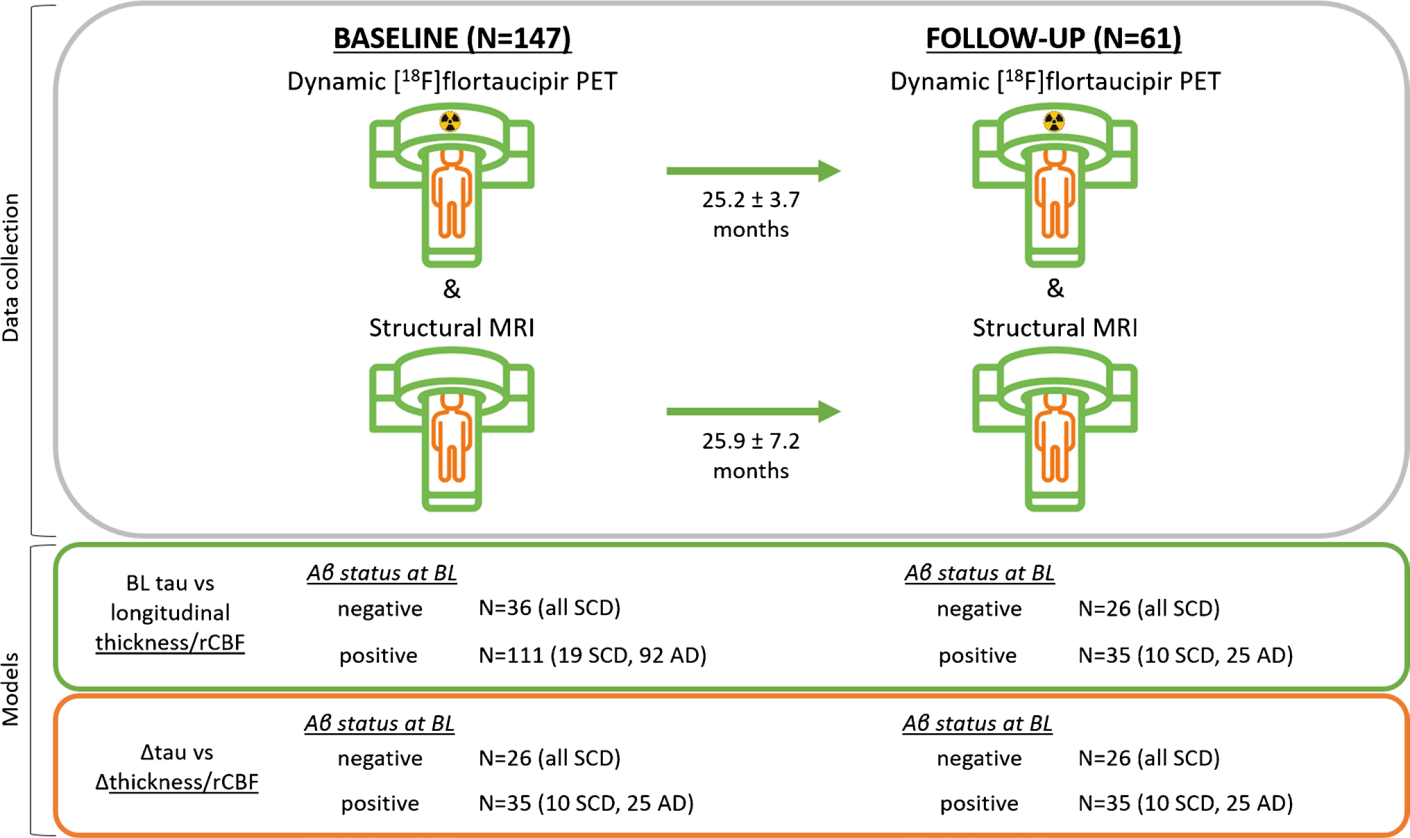
Overview of the study design. BL, baseline; rCBF, relative cerebral blood flow; Aβ, amyloid-β; SCD, subjective cognitive decline; AD, symptomatic Alzheimer’s disease

Out of the 61 individuals who underwent follow-up, 35 were Aβ+ and 26 Aβ− at baseline (Table 1, Fig. 1). Baseline demographics and characteristics of the total baseline sample (including the baseline-only sample, *n* = 147) are shown in sTable 1. Relative to the total baseline sample, the follow-up sample consisted of younger patients, with relatively higher MMSE scores and higher levels of global tau PET BP_ND_. In the follow-up sample, Aβ+ individuals were more often cognitively impaired (74% in Aβ+ vs 0% in Aβ− ; *p* < 0.001) and had lower MMSE scores when compared to Aβ− individuals (26 ± 3 in Aβ+ vs 29 ± 1 in Aβ− ; *p* < 0.001). Of the 61 individuals with longitudinal data, Aβ+ individuals had higher BP_ND_ values at both baseline and follow-up, whereas cortical thickness and R_1_ were lower (Table 1). Age, sex, and time between scans did not differ between Aβ+ and Aβ− individuals.

**Table 1:** Characteristics of the follow-up sample

	Total follow-up sample	Amyloid positive	Amyloid negative
Sample, *n*	61	35	26
Age, years	65.4 ± 7.4	66.3 ± 7.2	63.7 ± 7.7
Female, *n*	27	15	12
Cognitively impaired, *n*	26	26***	0
MMSE (baseline)	27 ± 3	26*** ± 3	29 ± 1
Time between MRI scans, minutes	25.9 ± 7.2	26.3 ± 5.0	25.3 ± 9.2
Time between PET scans, minutes	25.2 ± 3.7	25.8 ± 3.4	24.5 ± 4.0
Baseline global tau PET BP_ND_	0.13 ± 0.18	0.22*** ± 0.19	0.02 ± 0.03
Follow-up global tau PET BP_ND_	0.19^b^ ± 0.22	0.30***^b^ ± 0.23	0.04^b^ ± 0.03
Annual change global tau PET BP_ND_	0.02 ± 0.03	0.04*** ± 0.03	0.01 ± 0.01
Baseline global cortical thickness, mm	2.11 ± 0.07	2.08** ± 0.07	2.14 ± 0.07
Follow-up global cortical thickness, mm	2.09^a^ ± 0.09	2.05***^b^ ± 0.08	2.14 ± 0.07
Annual change global cortical thickness	−0.01 ± 0.02	−0.02** ± 0.02	0.00 ± 0.02
Baseline global *R*_1_	0.90 ± 0.05	0.88* ± 0.05	0.91 ± 0.04
Follow-up global *R*_1_	0.90 ± 0.05	0.88** ± 0.05	0.92 ± 0.05
Annual change global *R*_1_	−0.00 ± 0.01	−0.00 ± 0.02	0.00 ± 0.01

Amyloid + vs  − : *p < 0.05, **p < 0.01, ***p < 0.001; baseline vs follow-up: ^a^*p* < 0.01, ^b^*p* < 0.001

### Longitudinal change

**Fig. 2:**
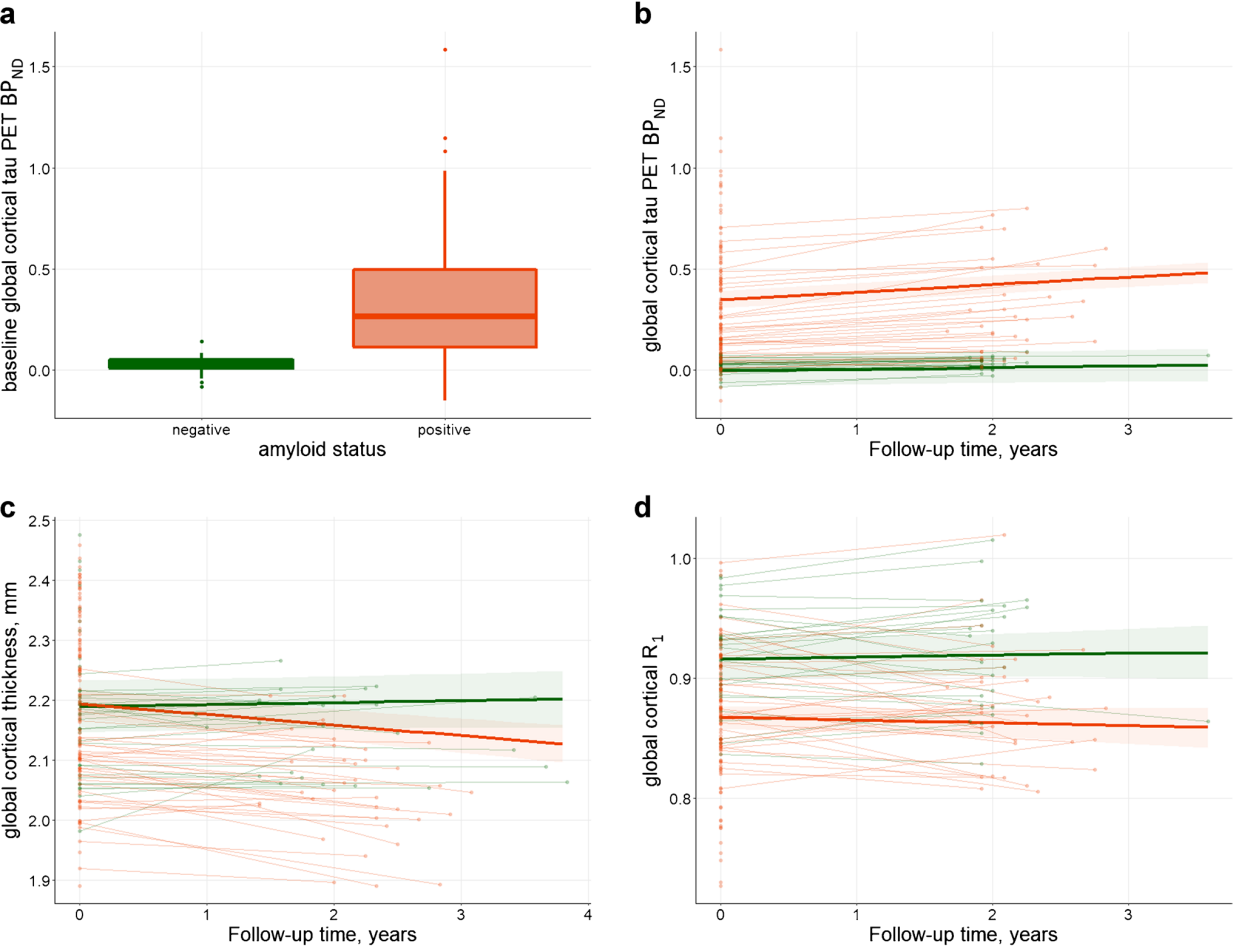
Plots showing baseline tau PET BP_ND_ (**a**) and longitudinal tau PET BP_ND_ (**b**), cortical thickness (**c**), and *R*_1_ (**d**) in amyloid negative (green) and positive (red) individuals

Tau PET BP_ND_ significantly increased over time in both Aβ− and Aβ+ individuals, with the largest increases observed in the Aβ+ individuals (Table 1, Fig. 2). Cortical thickness decreased over time in Aβ+ individuals only. rCBF, however, did not significantly change over time in either Aβ+ or Aβ− individuals (Table 1, Fig. 2). Although LMM results did not show a significant (average) change in rCBF on the group level, we did observe substantial changes at the individual level (both increases and decreases, potentially canceling each other out when calculating an effect over the whole group; Fig. 2). We therefore did perform the LMMs with longitudinal rCBF as dependent variable.

### Longitudinal cortical thickness as outcome measure

#### Baseline tau PET BP_ND_ as determinant

**Fig. 3:**
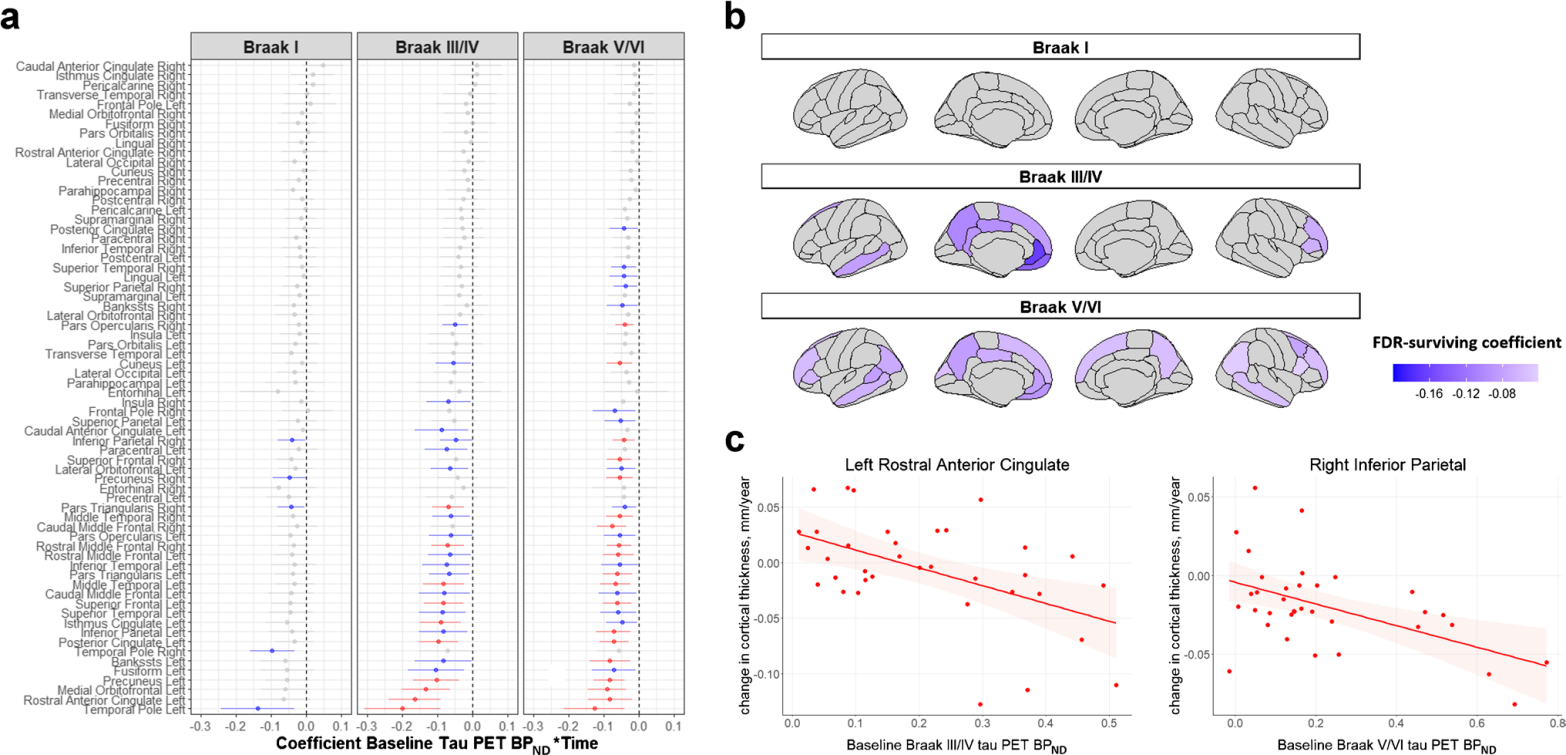
Association between baseline tau PET (BP_ND_) and longitudinal cortical thickness (mm) in amyloid-positive individuals. **a** Forest plot showing model estimates with 95% confidence intervals from linear mixed models with baseline tau PET BP_ND_ as determinant, longitudinal cortical thickness as outcome measure and age, sex, and time as covariates. Gray = non-significant. Blue = *p* < 0.05. Red = *p*_FDR_ < 0.05. **b** FDR-surviving results visualized using the ggseg R package. **c** Two scatterplots exemplifying FDR-surviving associations between baseline tau PET BP_ND_ and longitudinal cortical thickness

In Aβ+ individuals, linear mixed models showed that higher tau BP_ND_ at baseline was associated with cortical thinning over time (Fig. 3). More specifically, tau PET BP_ND_ in Braak III/IV was associated with a decrease in cortical thickness over time in widespread cortical regions, including parietal, (medial) frontal, and (lateral) temporal lobes. Comparable results were observed with tau PET BP_ND_ in Braak V/VI as the determinant, although with generally lower effect sizes compared to Braak III/IV. Tau PET BP_ND_ in Braak I showed only weak associations (not surviving FDR-correction) with cortical thinning over time. In Aβ− individuals, no FDR-surviving associations were found for any of the Braak ROIs (sFigure 1).

#### Annual change tau PET BP_ND_ as determinant

Linear mixed models did not yield any FDR-correction surviving associations between annual change in tau PET BP_ND_ and longitudinal cortical thickness in both Aβ+ and Aβ− individuals (sFigure 3).

### Longitudinal rCBF as outcome measure

#### Baseline tau PET BP_ND_ as determinant

Linear mixed models yielded no FDR-correction surviving associations between baseline tau PET BP_ND_ and longitudinal rCBF in neither Aβ+ nor Aβ− individuals (sFigure 2).

#### Annual change tau PET BP_ND_ as determinant

Linear mixed models showed that increases in tau PET BP_ND_ over time were associated with increases in R_1_ over time in the inferolateral and superior parietal gray matter in Aβ+ individuals (sFigure 4). No associations surviving correction for multiple comparisons were found in Aβ− individuals.

## Discussion

We assessed the associations between tau pathology and neuronal injury (reflected by measures of cortical thickness and rCBF) over time. We showed that higher tau pathology at baseline (especially in the Braak III/IV region) was associated with faster cortical thinning over time in Aβ+ individuals. Annual change in tau pathology did not show associations with cortical thinning over time, but larger increases in tau pathology did show associations with larger increases in *R*_1_ over time in inferolateral and superior parietal regions in Aβ+ individuals. Our results are in line with disease models proposing that tau load is a key driver of local cortical thinning and stress the need for future longitudinal studies into the role of rCBF in the pathophysiological process of AD. Furthermore, our results indicate that a single tau PET scan at baseline best predicts cortical thinning over time when compared to longitudinal tau PET imaging. This in turn highlights the potential of a single tau PET to improve the prognosis and selection of the right target population for clinical trials.

Our findings of higher tau pathology at baseline predicting cortical thinning over time in Aβ+ individuals are in line with previous studies, demonstrating local and non-local associations between tau pathology and longitudinal cortical thinning in Aβ+ individuals [2, 5, 11, 12, 40]. Regions most commonly showing associations between tau pathology and cortical thinning over time include frontotemporal and occipitoparietal regions [2, 11, 12, 40], which is similar to our findings, showing the strongest effects for Braak III/IV regional tau pathology and increased cortical thinning in the frontotemporal-parietal regions. We also assessed the association between (annual) change in tau pathology and (annual) change in cortical thickness. Whereas others reported (moderate) associations between larger increases in tau pathology and larger decreases in cortical thickness in individuals with mild cognitive impairment or atypical AD [12, 13], we found no associations surviving correction for multiple testing between change in tau pathology and longitudinal cortical thickness. A potential reason for these differences across studies might be the variance in parameters used. In our study, we used fully quantitative tau PET BP_ND_ to precisely measure (change in) tau pathology, whereas other studies used the semi-quantitative standardized uptake value ratio (SUVr), which is more sensitive to blood flow-introduced bias, specifically in longitudinal settings [41]. However, recently it has been demonstrated that SUVr provides an accurate estimate of specific binding for [^18^F]flortaucipir over a two-year follow-up during which changes in flow are small [42], making this unlikely to be a sole explanation for differences in results between studies. A difference in statistical power might also have played a role, since we did find some associations between larger increases in tau pathology with decreases in cortical thinning in Aβ+ individuals when using a more liberal statistical threshold (without correction for multiple testing). Taken together, in Aβ+ individuals we found robust associations between baseline tau pathology and longitudinal cortical thickness, while no association between change in tau pathology and longitudinal cortical thickness was found. This might be explained by the difference in sample size in the statistical models for baseline tau PET BP_ND_ (*n* = 147) vs annual change in tau PET BP_ND_ (*n* = 61) as determinant. It may also be that our follow-up sample was somewhat biased, as the most advanced individuals might have dropped out more frequently. Another reason may be that there is a temporal delay for the neurotoxic effects of tau to manifest, making baseline tau pathology more important for the occurrence of neurodegeneration when compared to change in tau. Lastly, the magnitude of annual change in tau PET BP_ND_ is generally modest, leading to a difference in variability which may affect the ability of finding statistically significant effects.

Our results demonstrated an increase in tau pathology over time, irrespective of baseline amyloid status. Although current hypothetical models propose that amyloidosis is an upstream driver of tau accumulation [4, 43, 44], and tau pathology is generally only found to be accumulating in Aβ+ individuals, there are some studies showing significant cortical tau accumulation in Aβ− individuals [40, 45]. Accumulation of tau pathology in Aβ− individuals may be driven by processes related to aging, since positive associations between rates of tau accumulation and age were found among cognitively unimpaired Aβ− individuals [44]. It could also be that Aβ− individuals with accumulating tau pathology do actually have amyloid pathology, but at subthreshold or below detection levels, as it has previously been shown that in individuals who were nominally Aβ− , both the rate of Aβ accumulation and the baseline Aβ load predicted tau deposition in cortical Braak regions associated with AD [46, 47]. Future studies into longitudinal tau accumulation in the context of amyloid pathology may therefore consider looking at continuous amyloid levels rather than binary amyloid status.

Relative cerebral blood flow did not change over time during the two-year follow-up period. Although longitudinal changes in rCBF have not been studied previously using [^18^F]flortaucipir *R*_1_ in other cohorts, we might compare our findings with studies investigating CBF using SPECT, ^15^O-H_2_O PET, and MRI. Previous findings were indicative of both increases and decreases in rCBF over time in individuals without dementia who had high-amyloid load [24] and decreases in (fast progressing) AD patients [48]. These findings are in contrast with the lack of change over time in our study and also in contrast with our hypothesis, where we assumed that CBF alterations occur in between tau accumulation and atrophy in the pathophysiological development of AD, thus expecting changes in rCBF to occur, especially when changes in cortical thickness are observed. One explanation for this discrepancy might be that changes in rCBF occur in different directions (in- or decreases) on the individual level (as can also be observed in Fig. 2), potentially canceling each other out, leading to absence of average effects or change on the group level. Another explanation may lie in results of another study assessing the relationship between longitudinal perfusion measures and tau pathology (as measured with PET), as they found a lack of overlap between declining perfusion and increases in tau pathology, suggesting a lag phase between these two processes [49]. This may also be the case here, given that tau pathology did change over time, while R_1_ did not (yet) in our study. Lastly, an explanation might lie in the composition of our Aβ+ group. Some literature describes increases in CBF over time in individuals without dementia with low-, intermediate- or high amyloid load [23, 24]. This might suggest that the increase in CBF represents a compensatory mechanism in response to first-occurring pathology. The fact that our amyloid-positive group also included non-demented individuals, in whom this compensatory increase in CBF potentially takes place, might have contributed to the positive association between increases in Braak III/IV tau pathology and parietal CBF as found in our study.

A strength of the current study is that fully dynamic [^18^F]flortaucipir PET data was used to obtain quantitatively accurate measures of both tau pathology and rCBF in a sample covering the whole AD spectrum (CN-dementia). Furthermore, relative to previous studies this study had a long follow-up period of 25 months. Some caveats are, however, to be considered in the interpretation of our results. First, three different atlases were used to process our data, i.e., Hammers (Braak III–VI) and Svarer (Braak I) for PET and Desikan-Killiany for MRI. We opted not to change our well-established PET pipeline and accept the inherent variations in ROI definitions introduced by this methodological decision. Second, the subset of individuals with longitudinal tau PET data available was relatively small. This may have reduced the statistical power to detect effects. Also, there was likely a bias in our follow-up sample, where older patients and patients with lower MMSE scores dropped out more frequently. This bias is unfortunately common in longitudinal AD studies and likely excludes more progressed AD patients at follow-up. Furthermore, our sample is relatively young and findings might not translate to older patient populations where co-pathologies (independently of tau pathology) associated with brain atrophy are more common. Last, some of the regions that yielded significant associations, like the temporal poles or orbitofrontal cortices, are known to be susceptible to Freesurfer segmentation errors. However, these regions are found repeatedly throughout the literature and image segmentations were thoroughly checked prior to analyses.

In conclusion, we assessed the association between i) baseline and ii) change in tau pathology with longitudinal atrophy and rCBF by using dynamic [^18^F]flortaucipir PET and structural MRI scans. We demonstrate that tau pathology accumulated in individuals, irrespective of their baseline amyloid status and that cortical thickness decreases over time in Aβ+ individuals only. On group level no change in rCBF over the two-year follow-up period was observed, but both in- and decreases were found on the individual level. This stresses the need for future longitudinal studies into complex longitudinal changes in rCBF and their association with (changes in) tau pathology. Furthermore, higher tau pathology at baseline was associated with faster cortical thinning over time in Aβ+ individuals. These results support disease models in which tau pathology is a driver of neurodegenerative processes, which will further contribute to potentially establishing the utility of (a single) tau PET as a predictive tool in terms of identifying slow- or fast-degenerating individuals, which may in turn be important for selection criteria in clinical trials.

## Supplementary Information

Below is the link to the electronic supplementary material.

Supplementary file1 (DOCX 6.78 MB)

## Data Availability

Data can be made available upon reasonable request.
