# Supplementary material for: Tau pathology as determinant of changes in atrophy and cerebral blood flow: a multi-modal longitudinal imaging study
Source: Eur J Nucl Med Mol Imaging. 2023 Mar 28;50(8):2409–19. doi: 10.1007/s00259-023-06196-2 (PMC10250461; doi:10.1007/s00259-023-06196-2)

**SUPPLEMENT**

**Tau pathology as determinant of changes in atrophy and cerebral blood flow: a multi-modal longitudinal imaging study**

Denise Visser^1,2,3^, Sander C.J. Verfaillie^1,2,3,4^, Iris Bosch^1,3,5,6^, Iman Brouwer^1,2,3^, Hayel Tuncel^1,2,3^, Emma M. Coomans^1,2,3^, Roos M. Rikken^1,2,3^, Sophie E. Mastenbroek^1,2,3,7^, Sandeep S.V. Golla^1,2,3^, Frederik Barkhof^1,2,3,8^, Elsmarieke van de Giessen^1,2,3^, Bart N.M. van Berckel^1,2,3^, Wiesje M. van der Flier^2,9,10^, Rik Ossenkoppele^2,7,9^

*^1^ Radiology & Nuclear Medicine, Vrije Universiteit Amsterdam, Amsterdam UMC, Amsterdam, The Netherlands*

*^2^ Amsterdam Neuroscience, Neurodegeneration, Amsterdam, The Netherlands*

*^3^* *Amsterdam Neuroscience, Brain Imaging, Amsterdam, The Netherlands*

*^4^* *Amsterdam UMC location University of Amsterdam, Medical Psychology, Meibergdreef 9, Amsterdam, the Netherlands*

*^5^ Department of Psychiatry and Neurochemistry, Institute of Neuroscience and Physiology, The Sahlgrenska Academy, University of Gothenburg, Gothenburg, Sweden*

*^6^ Wallenberg Centre for Molecular and Translational Medicine, University of Gothenburg, Gothenburg, Sweden*

*^7^ Clinical Memory Research Unit, Lund University, Lund, Sweden*

*^8^ Institutes of Neurology and Healthcare Engineering, University College London, London, UK*

*^9^ Alzheimer Center Amsterdam, Neurology, Vrije Universiteit Amsterdam, Amsterdam UMC location VUmc, Amsterdam, The Netherlands*

*^10^ Department of Epidemiology and Data Science, Vrije Universiteit Amsterdam, Amsterdam UMC, Amsterdam, The Netherlands*

Corresponding author:

Denise Visser, Department of Radiology & Nuclear Medicine, Amsterdam Neuroscience, Vrije Universiteit Amsterdam, Amsterdam UMC, Amsterdam, The Netherlands, P.O. Box 7057, 1007 MB Amsterdam, The Netherlands; E-mail: d.visser2@amsterdamumc.nl; ORCID ID: 0000-0002-3642-146X

Rik Ossenkoppele, Alzheimer Center Amsterdam, Department of Neurology, Amsterdam Neuroscience, Vrije Universiteit Amsterdam, Amsterdam UMC, Amsterdam, The Netherlands | Clinical Memory Research Unit, Lund University, Lund, Sweden, E-mail: [r.ossenkoppele@amsterdamumc.nl](mailto:r.ossenkoppele@amsterdamumc.nl); ORCID ID: 0000-0003-1584-7477

**sTable 1. Characteristics of the total sample at baseline**

|  | **Total sample** | **Follow-up subset** |
| --- | --- | --- |
| **Sample, n** | 147 | 61 |
| **Age, y** | 65.4 ± 7.4 | 65.1 ± 7.5 |
| **Female, n (%)** | 70 (48%) | 27 (44%) |
| **Cognitively impaired, n** | 92 | 26 |
| **Amyloid positive, n** | 111 | 35 |
| **MMSE** | 26 ± 4 | 27 ± 3 |
| **Time between MRI scans, m** | - | 25.9 ± 7.2 |
| **Time between PET scans, m** | - | 25.2 ± 3.7 |
| **Global tau PET BP_ND_** | 0.26 ± 030 | 0.13 ± 0.18 |
| **Global cortical thickness, mm** | 2.19 ± 0.13 | 2.11 ± 0.07 |
| **Global R_1_** | 0.88 ± 0.05 | 0.90 ±0.05 |

**sFigure 1. Association between baseline tau PET (BP_ND_) and longitudinal cortical thickness (mm) in amyloid negative individuals. a)** Forest plot showing results from linear mixed models with baseline tau PET BP_ND_ as determinant, longitudinal cortical thickness as outcome measure and age, sex and time as covariates. Grey = non-significant. Blue = p<0.05 (not surviving FDR-correction).
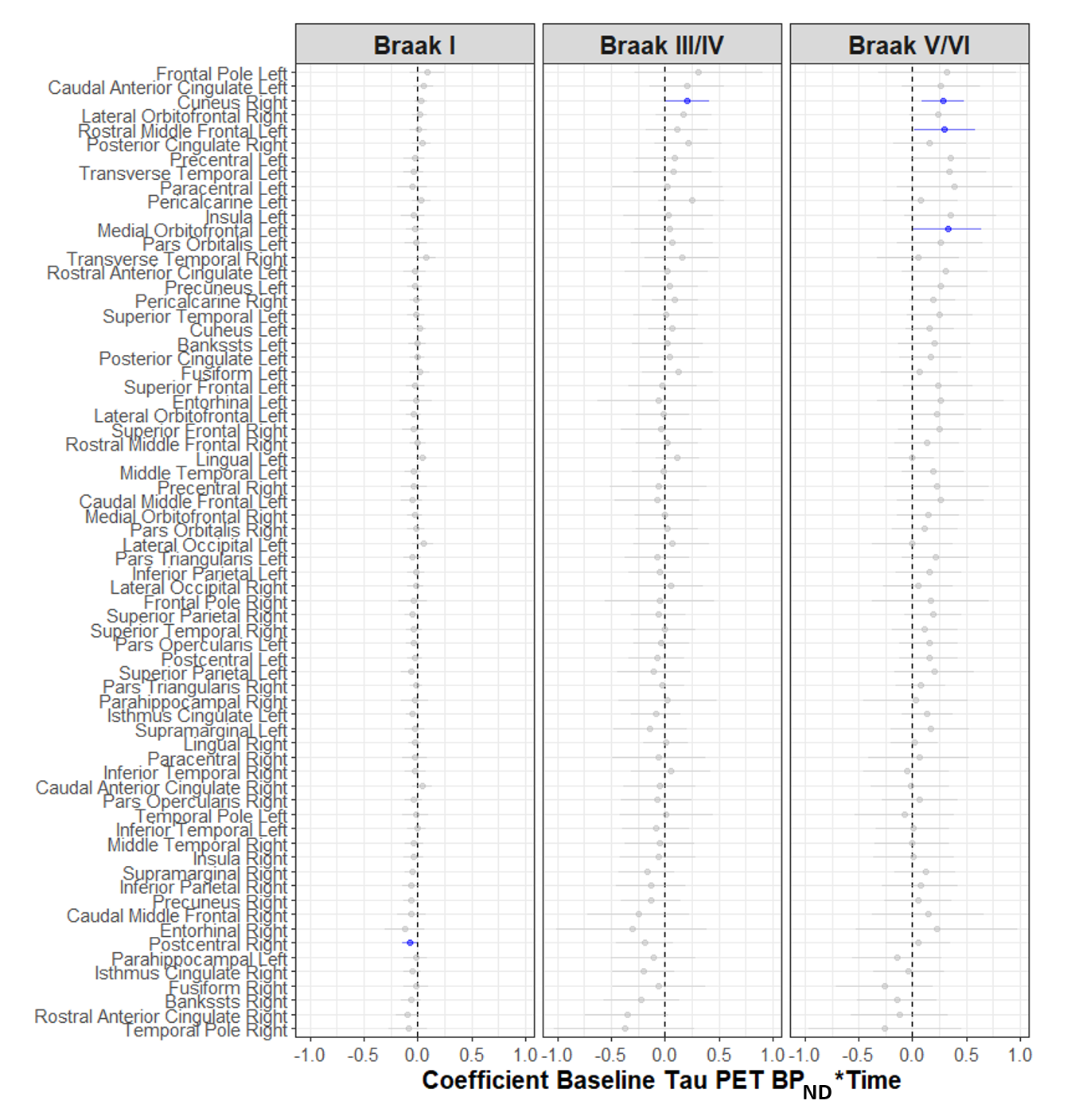


**sFigure 2. Association between baseline tau PET (BP_ND_) and longitudinal rCBF (R_1_) in a) amyloid negative and b) amyloid positive individuals.** Forest plot showing model estimates with 95% confidence intervals from linear mixed models with baseline tau PET BP_ND_ as determinant, longitudinal rCBF (R_1_) as outcome measure and age, sex and time as covariates. Grey = non-significant. Blue = p<0.05 (not surviving FDR-correction).


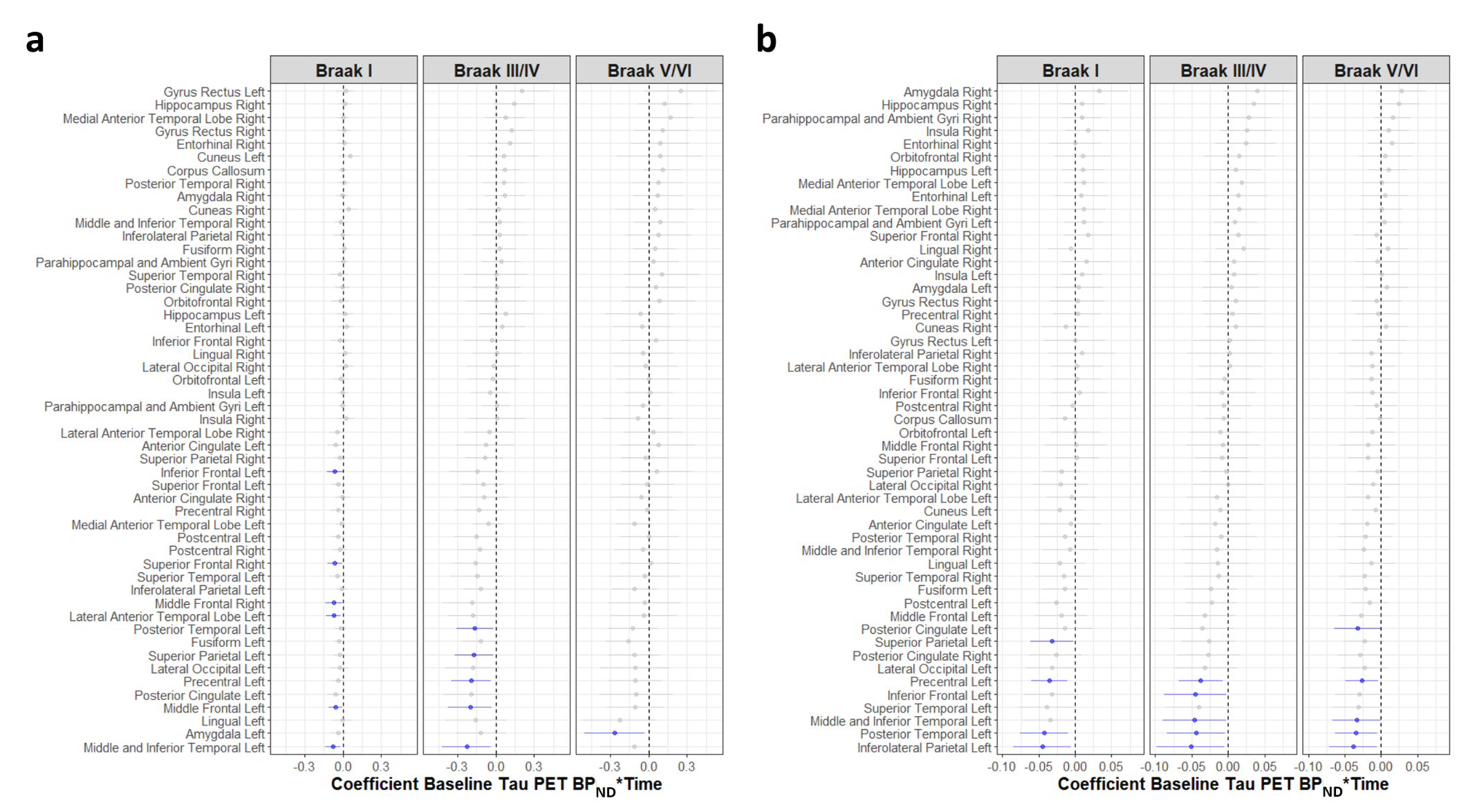


**sFigure 3.** **Association between annual change in tau PET (BP_ND_/year) and longitudinal cortical thickness in a) amyloid negative and b) amyloid positive individuals.** Forest plot showing model estimates with 95% confidence intervals from linear mixed models with annual change in tau PET BP_ND_ as determinant, longitudinal cortical thickness as outcome measure and age, sex, time and baseline tau PET BP_ND_ as covariates. Grey = non-significant. Blue = p<0.05 (not surviving FDR-correction).
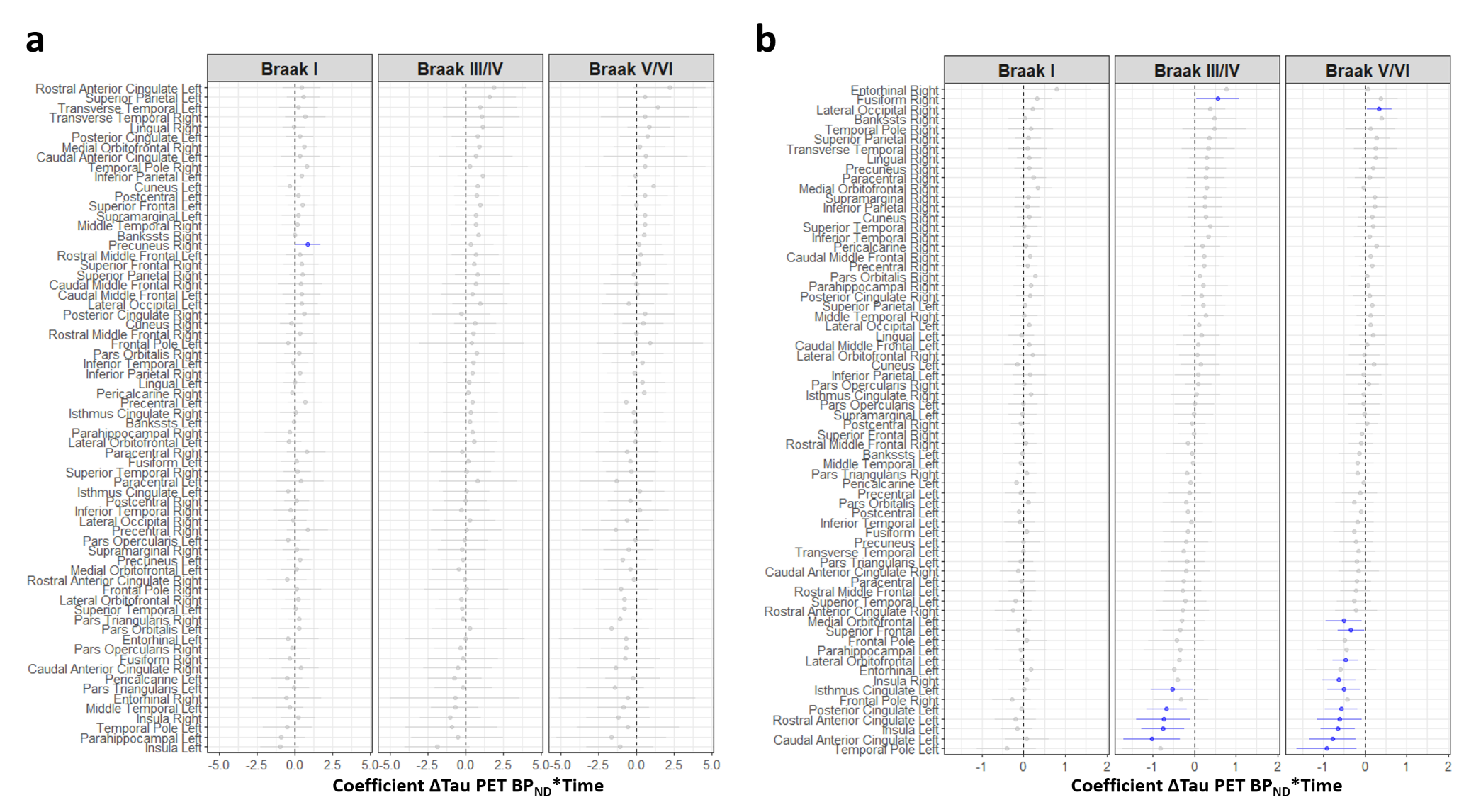


**sFigure 4.** **Association between annual change in tau PET (BP_ND_/year) and longitudinal R_1_ in a) amyloid negative and b) amyloid positive individuals.** Forest plot showing model estimates with 95% confidence intervals from linear mixed models with annual change in tau PET BP_ND_ as determinant, longitudinal rCBF (R_1_) as outcome measure and age, sex, time, and baseline R_1_ as covariates. Grey = non-significant. Blue = p_uncorrected_<0.05. Red = p_FDR_<0.05.


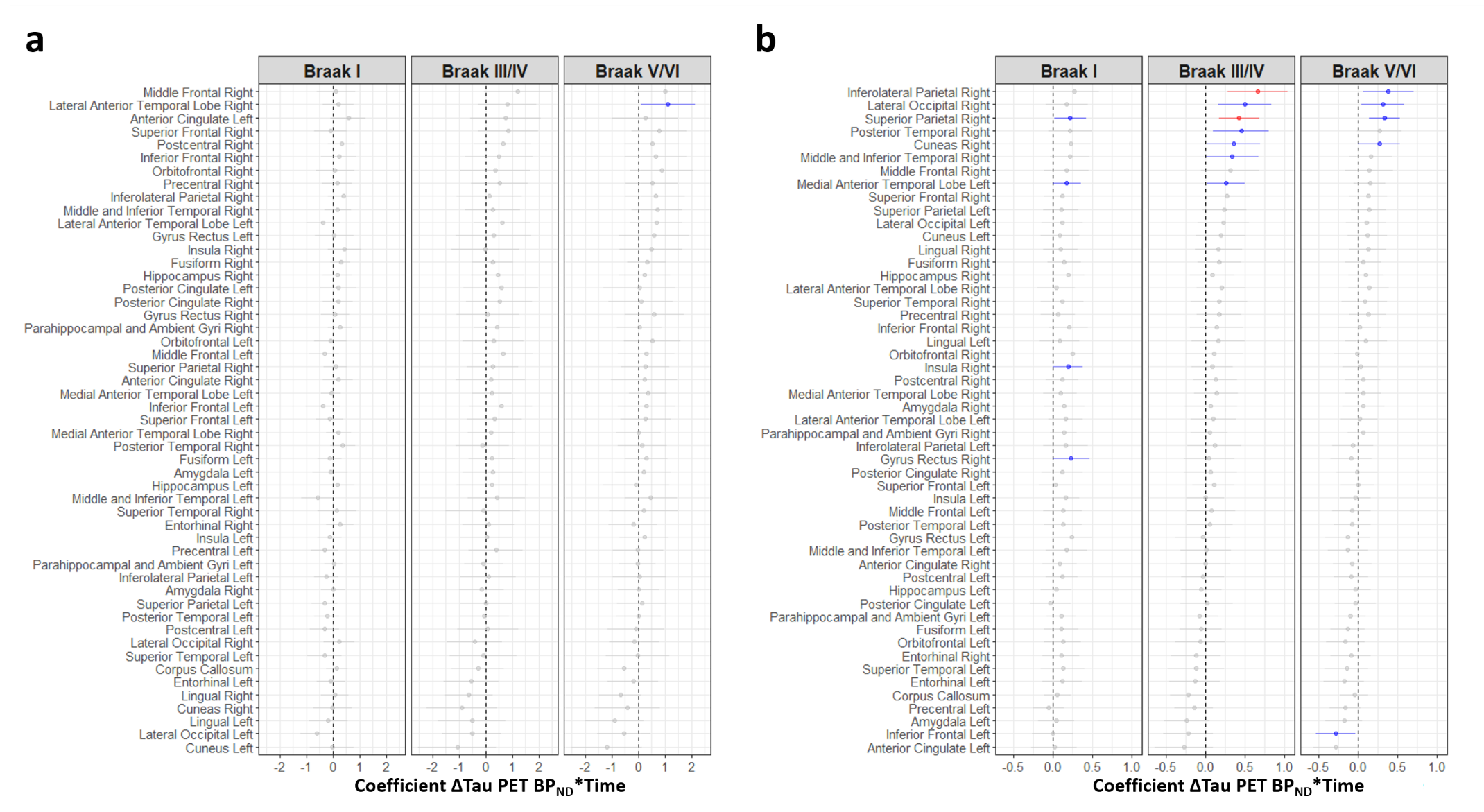

Supplement: Supplementary file 1 — Supplementary file1 (DOCX 6.78 MB) [file 259_2023_6196_MOESM1_ESM.docx]
